# CT texture analysis and node-RADS CT score of mediastinal lymph nodes - diagnostic performance in lung cancer patients

**DOI:** 10.1186/s40644-022-00506-x

**Published:** 2022-12-26

**Authors:** Hans-Jonas Meyer, Benedikt Schnarkowski, Johanna Pappisch, Teresa Kerkhoff, Hubert Wirtz, Anne-Kathrin Höhn, Sebastian Krämer, Timm Denecke, Jakob Leonhardi, Armin Frille

**Affiliations:** 1 Department of Diagnostic and Interventional Radiology, University of Leipzig, Leipzig, Germany; 2 Department of Respiratory Medicine, University Hospital Leipzig, University of Leipzig, Leipzig, Germany; 3 Department of Pathology, University Hospital Leipzig, University of Leipzig, Leipzig, Germany; 4 Department of Thoracic Surgery, University Hospital Leipzig, University of Leipzig, Leipzig, Germany; 5 Integrated Research and Treatment Centre (IFB) Adiposity Diseases, University Medical Centre Leipzig, Leipzig, Germany

**Keywords:** Computed tomography, Lymph node, Lung cancer, Texture

## Abstract

**Background:**

Texture analysis derived from computed tomography (CT) can provide clinically relevant imaging biomarkers. Node-RADS is a recently proposed classification to categorize lymph nodes in radiological images. The present study sought to investigate the diagnostic abilities of CT texture analysis and Node-RADS to discriminate benign from malignant mediastinal lymph nodes in patients with lung cancer.

**Methods:**

Ninety-one patients (*n* = 32 females, 35%) with a mean age of 64.8 ± 10.8 years were included in this retrospective study. Texture analysis was performed using the free available Mazda software. All lymph nodes were scored accordingly to the Node-RADS classification. All primary tumors and all investigated mediastinal lymph nodes were histopathologically confirmed during clinical workup.

**Results:**

In discrimination analysis, Node-RADS score showed statistically significant differences between N0 and N1-3 (*p* < 0.001). Multiple texture features were different between benign and malignant lymph nodes: S(1,0)AngScMom, S(1,0)SumEntrp, S(1,0)Entropy, S(0,1)SumAverg. Correlation analysis revealed positive associations between the texture features with Node-RADS score: S(4,0)Entropy (*r* = 0.72, *p* < 0.001), S(3,0) Entropy (*r* = 0.72, *p* < 0.001), S(2,2)Entropy (*r* = 0.72, *p* < 0.001).

**Conclusions:**

Several texture features and Node-RADS derived from CT were associated with the malignancy of mediastinal lymph nodes and might therefore be helpful for discrimination purposes. Both of the two quantitative assessments could be translated and used in clinical routine.

## Introduction

Texture analysis is an emergent research field to provide novel quantitative biomarkers derived from radiological images [1–7]. Various applications of texture analysis have been investigated throughout clinical medicine, predominantly in the field of oncology [1–7]. Different spatial characteristics were used for better discrimination purposes, treatment prediction and prognosis stratification in several tumor entities. In short, texture analysis derived from radiological images can provide quantitative information beyond the scope of the radiologist’s clinical observation [1–7].

Node-RADS (Node-Reporting and Data System) is a recently proposed classification system to standardize the clinically reporting for lymph nodes [8]. By means of the two categories “size” and “configuration”, a 5-point probability score for malignancy ranging from 1 (“very low likelihood”) to 5 (“very high likelihood”) is assigned [8]. However, Node-RADS classification system has not yet been validated in the clinical routine. For other standardized reporting systems in radiology, such as BI-RADS (breast imaging RADS), PI-RADS (prostate imaging RADS), LI-RADs (liver imaging RADS), TI-RADS (thyroid imaging RADS), a plethora of studies have attributed benefits in the establishment of tumor diagnosis and evaluation of malignancy probability [9–12]. For PI-RADS, even an association with the important histopathologic Gleason score indicates the direct association with tumor aggressiveness [13]. Presumably, possible associations between radiological classification systems with the underlying pathobiology of the tumors can be further identified.

For mediastinal lymph nodes a cut-off value of 10 mm in short axis diameter is reported to discriminate benign from malignant lymph nodes, which could yield a sensitivity of 55% and a specificity of 81% [14]. Fluorodeoxyglucose positron emission tomography with combined computed tomography (FDG-PET/CT) utilizing the metabolic rate in malignant tissue has shown to facilitate superior diagnostic accuracy with a sensitivity of 77% and a specificity of 86% [14].

For histopathological examination, biopsy of mediastinal lymph nodes can be obtained by conventional endobronchial ultrasound transbronchial needle aspiration (EBUS-TBNA) and mediastinoscopy. Both modalities have been shown to achieve a sensitivity of 61–65 and 79%, respectively, with a specificity for both of nearly 100% [15–17].

However, there is to date no study investigating the diagnostic potential of Node-RADS in oncologic imaging beyond the initial landmark paper [8]. Few but promising results were published regarding the diagnostic benefit of texture analysis for mediastinal lymph nodes [18–21].

In the light of novel medical treatment options for and more and more complex categorizations of lung cancer patients, texture analysis of mediastinal lymph nodes seems of particular clinical interest and relevance [22–24]. Quantitative imaging should aid in the complex clinical decision-making process in patient with lung cancer.

Therefore, the purpose of the present study was to investigate whether CT-derived texture analysis parameters and Node-RADS categorization of mediastinal lymph nodes can improve the diagnostic performance for dignity in lung cancer patients.

## Methods

### Study design

This retrospective, observational study involving human participants was performed in accordance with the ethical standards of the institutional and/or national research committee and with the 1964 Helsinki declaration and its later amendments or comparable ethical standards.

It received ethical approval from the local ethics committee at the Medical Faculty of Leipzig University (IRB00001750, AZ: 259/18-ek).

Standardized clinical, pathological (histology, tumor stage) and survival data of patients with lung cancer (ICD-10 C34*) who were diagnosed at the University Hospital Leipzig were derived from the regional, clinical cancer registry (Klinisches Krebsregister Leipzig).

The university hospital’s radiological database was retrospectively screened between January 2012 and December 2015 for patients with sufficient imaging data.

Inclusion criteria consisted of sufficient presurgical or prebiopsy CT images, histopathologically confirmed primary lung cancer and histopathological mediastinal lymph node analysis. The CT scans were acquired within 1 month before the invasive staging. All patients were finally pathologically staged according to the then valid 7th lung cancer TNM classification and staging system jointly published by Union Internationale Contre le Cancer (UICC) and the International Association for the Study of Lung Cancer (IASLC) [25].

Overall, 91 patients (*n* = 32 females, 35%) with a mean age of 64.8 ± 10.8 years were included in the analysis. An overview of the descriptive statistics of the included patients is given by Table 1.

**Table 1:** Demographic characteristics of the investigated patient sample

Parameter	N0	N1-3	***p***-value
**Age (y)**	64.2 ± 11	65.8 ± 11	0.66
**Gender (male, n, %)**	37 (65.5)	22 (62.8)	0.28
**Primary tumor**			
Adenocarcinoma	30	18	0.84
Squamous cell carcinoma	11	13	0.07
Large cell neuroendocrine carcinoma	6	3	0.95
Small cell carcinoma	1	1	
Typical carcinoid	4	0	
Atypical carcinoid	2	0	
Solid carcinoma, NOS	2	0	
**Grading**			
0	4	0	0.11
1	16	2	0.008
2	18	12	0.83
3	15	22	0.007
4	1	1	0.98
**pT**			
0	1	0	0.43
1	36	12	0.006
2	12	10	0.44
3	6	10	0.03
4	1	3	0.13
**pM**			
0	53	28	0.03
1a	1	2	0.31
1b	2	5	0.06
**Stage (UICC)**			
IA	37	0	< 0.001
IB	7	0	0.03
IIA	3	10	0.002
IIB	3	5	0.954
IIIA	1	13	< 0.001
IIIB	0	2	0.072
IVA	1	2	0.31
IVB	2	5	0.063
**Size of investigated lymph node** (short axis diameter in mm)	7.6 ± 1.9	13.2 ± 5.0	< 0.001

Abbreviation: *NOS* not other specified

### Imaging technique

CT was performed with a 128-slice CT scanner (Ingenuity 128, Philips, Hamburg, Germany). In *n* = 38 cases (42% of patients), intravenous iodine-based contrast medium (60 mL Imeron 400 MCT, Bracco Imaging Germany GmbH, Konstanz, Germany) was injected at a rate of 4.0 mL/s via a peripheral venous line. Automatic bolus tracking was performed in the aorta descenders with a trigger of 100 Hounsfield units (HU). Typical imaging parameters were: 100 kVp; 125 mAs; slice thickness,1 mm; pitch, 0.9.

### Texture analysis

CT images were processed with the freely available texture analysis software MaZda (version 4.7, available at http://www.eletel.p.lodz.pl/mazda/) [26, 27]. A polygonal region of interest (ROI) was placed on the largest, representative slide of the suspicious mediastinal lymph node. The ROI’s diameter was adjusted to the boundary of the lymph node. The measurement was performed in a blinded manner to the clinical and histopathological results by a resident of radiology (J.L.) with 3 years of general experience. For each ROI, gray-level (μ) normalization was performed, using the limitation of dynamics to μ ± 3 standard deviations to reduce the contrast and brightness variation, as previously performed [28, 29].

The extracted features were as follows: gray-level histogram co-occurrence matrix [angular second moment, contrast, correlation, entropy, sum entropy, sum of squares, sum average, sum variance, inverse difference moment, difference entropy, difference variance (for four directions), run-length matrix (run-length non-uniformity, gray-level non-uniformity, long run emphasis, short run emphasis, fraction of image in runs)], absolute gradient, autoregressive model (theta 1 to 4, sigma), and wavelet transform. In total, 279 texture features were retrieved for every patient.

**Fig. 1:**
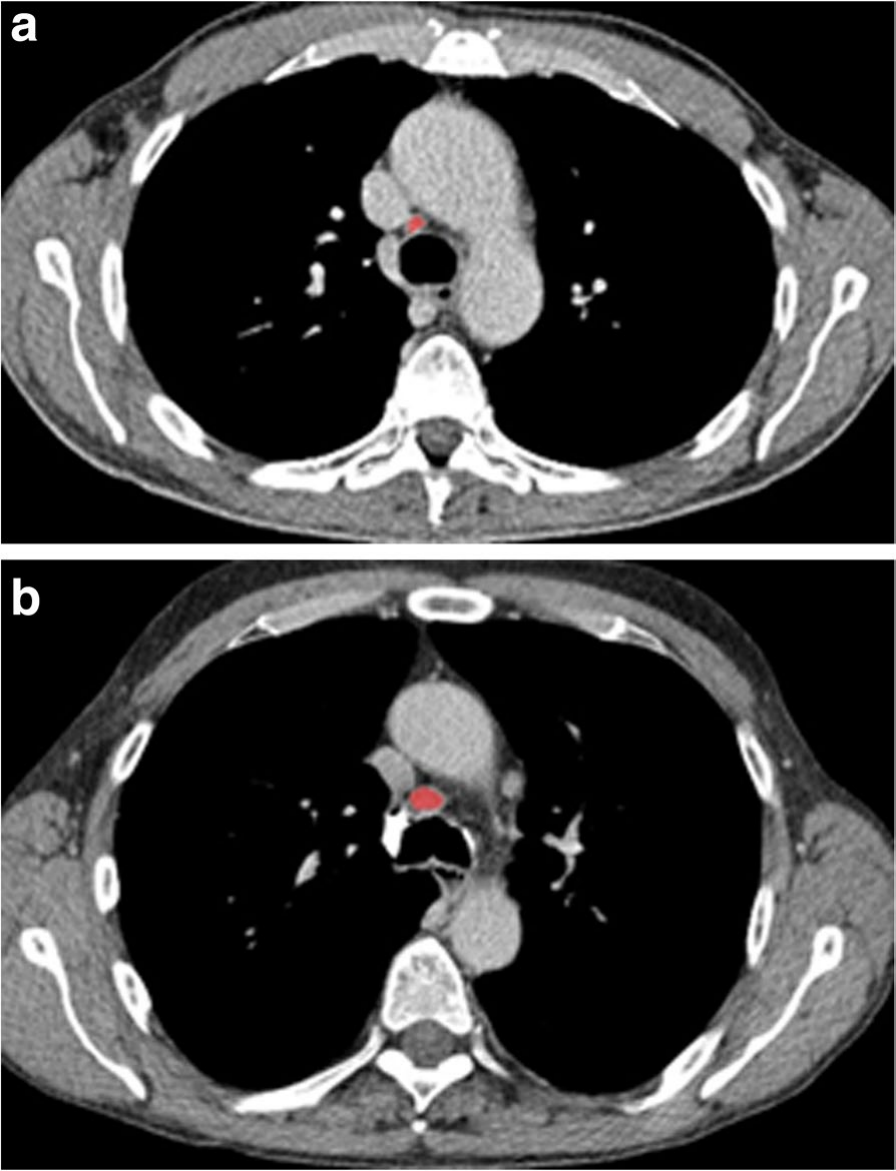
Representative cases of the patient sample. The mediastinal lymph node is highlighted in red, which was also the region of the interest for the texture analysis. **a** UICC IA, N0, Node-RADS category of 0, short-axis-diameter of 6 mm. **b** UICC IIA, N1, Node-RADS category of 3, short-axis-diameter of 11 mm

Figure 1 displays 2 representative cases of the patient sample for illustration purposes.

### Lymph node score

Suspicious mediastinal lymph nodes were scored according to the previously reported Node-RADS classification [8]. Scoring was performed by two radiologists with 3 years (reader one) and two 2 years (reader two) of experience in CT imaging analysis. In short, the classification categories range between 1 and 5, reflecting the level of probability of malignancy: “1—very low”; “2—low”; “3—equivocal”; “4—high”; “5—very high. Two main imaging findings were assessed: size and configuration. The lymph node’s size was classified as enlarged, when the short axis was above 10 mm. For configuration purposes, the texture was designated as either homogenous, heterogenous, focal or gross necrosis. The border was defined as smooth or irregular. The shape was defined as kidney bean with fat hilus or spherical without fat hilus. Both features resulted in the final lymph node category.

### Statistical analysis

The statistical analysis and graphics creation were performed with SPSS STATISTICS (IBM, Version 25.0; Armonk, NY, USA). Collected data were evaluated by means of descriptive statistics (absolute and relative frequencies). Spearman’s correlation coefficient (r) was used to analyze associations between investigated scores. Group differences were calculated with Mann-Whitney test for continuous data and Fisher exact test for categorical data. Receiver-operating characteristics (ROC) curve analysis was used to test for diagnostic accuracy. Inter-reader agreement was assessed with Cohen’s kappa. In all instances, two-sided *p*-*v*alues < 0.05 indicated statistical significance.

## Results

Table 1 displays the demographics of the investigated patients.

### Discrimination analysis of node-RADS score for N stage

Node-RADS scoring resulted for reader 1 in a total of *n* = 40 for Node-RADS 1 (44.0%), *n* = 21 for Node-RADS 2 (23.1%), *n* = 17 for Node-RADS 3 (18.6%), *n* = 5 for Node-RADS 4 (5.5%) and *n* = 8 (8.8%) for Node-RADS 5.

For reader 2, the results were *n* = 45 for Node-RADS 1 (49.5%), *n* = 21 for Node-RADS 2 (23.1%), *n* = 11 for Node-RADS 3 (12.1%), *n* = 5 for Node-RADS 4 (6.6%) and *n* = 8 (8.8%) for Node-RADS 5.

Inter-reader agreement was only moderate for the Node-RADS scoring (k = 0.48).

Distribution of malignancy according to each Node-RADS score for both readers are shown in Table 2.

**Table 2:** Malignancy rates according to Node-RADS score

Node-RADS score	Reader	Number of cases scored	Histopathologically confirmed malignancy
**1**	1	40	0 (0%)
	2	45	4 (8.8%)
**2**	1	21	9 (42.8%)
	2	21	9 (42.8%)
**3**	1	17	13 (76.5%)
	2	11	8 (72.7%)
**4**	1	5	5 (100%)
	2	6	6 (100%)
**5**	1	8	8 (100%)
	2	8	8 (100%)

For reader 1, Node-RADS 1 had a malignancy rate of 0% and for reader 2 8.8%, Node-RADS 2 a malignancy rate of 42.8% for both readers, Node-RADS 3 a malignancy rate of 76.5% for reader 1 and 72.7% for reader 2. Node-RADS 4 and 5 both had a malignancy rate of 100% for both readers.

In discrimination analysis, the total Node-RADS score showed statistically significant differences between N0 and N1-3 stage (for reader 1: mean 1.4 ± 0.6 score for N0 versus 3.3 ± 1.1 score for N1-3, for reader 2: 1.3 ± 0.6 score for N0 versus 3.1 ± 1.4 score for N1-3, *p* < 0.001, respectively).

**Fig. 2:**
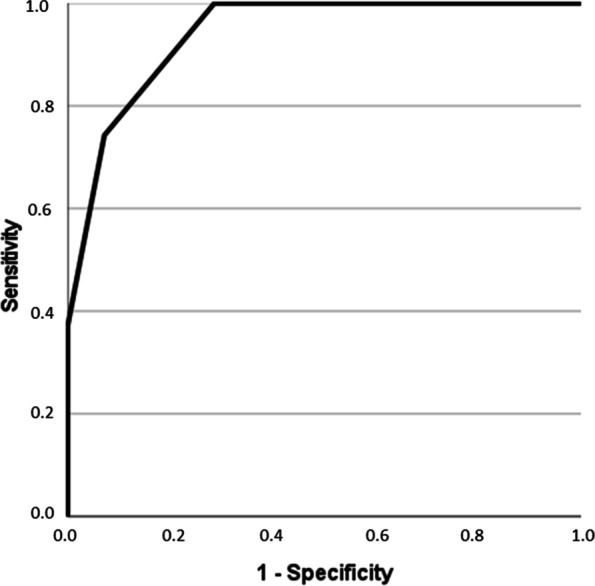
Result of the ROC curve analysis for discrimination of N0 versus N1-3 with total Node-RADS-Score with an AUC of 0.94. A threshold value of 2 resulted in a sensitivity of 0.74 and a specificity of 0.93

ROC curve analysis for lymph node discrimination (N0 versus N1-3) showed an area under the curve (AUC) of 0.94. A threshold value of 2 resulted in a sensitivity of 0.74 and a specificity of 0.93 (Fig. 2).

The underlying Node-RADS subcategory size, as represented by short axis diameter, reached statistically significant difference between N0 and N1-3 stage (mean 7.6 ± 1.9 mm for N0 versus 13.2 ± 5.0 mm for N1-3, *p* < 0.001). Also, for both readers, the Node-RADS subcategories texture (*p* < 0.001), border (*p* < 0.001) and shape (*p* < 0.001) reached statistical significance with higher subcategory scores correlating with higher likelihood of positive N-stages.

**Fig. 3:**
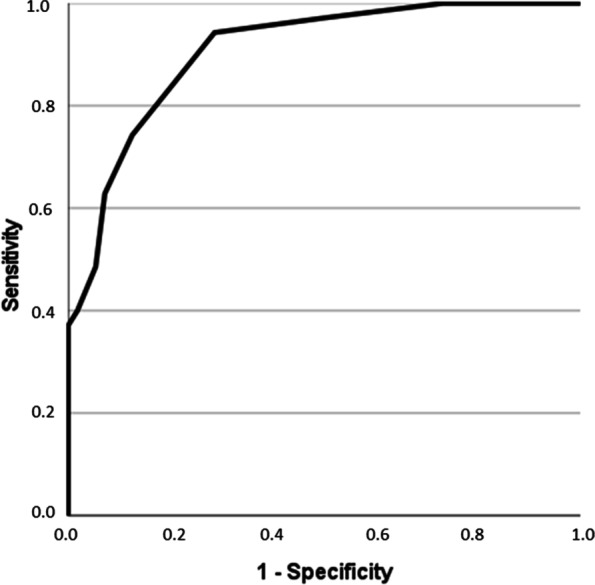
Results of the ROC curve analysis for discrimination of N0 vs N1-3 with texture features and short axis diameter. A threshold value of 10 mm was selected and resulted in an AUC of 0.91 with a sensitivity of 0.74 and a specificity of 0.88

When performing ROC curve analysis for lymph node discrimination (N0 versus N1-3) using the short axis diameter; a threshold value of 10 mm was selected and resulted in an AUC of 0.91 with a sensitivity of 0.74 and a specificity of 0.88 (Fig. 3).

No significant differences were found between the AUC for lymph node discrimination and the AUC for discrimination employing total Node-RADS score or short axis diameter, respectively (*p* = 0.178).

### Discrimination analysis of texture parameters for N stage

A total of 133 parameters showed statistically significant differences between the two groups, especially of the second order-group. In all, 86 features reached statistically significant *p*-values (< 0.001, Table 3).

**Table 3:** Texture features showing statistically significant differences between lymph nodes N0 versus lymph nodes N1-3 with *p*-values < 0.001

Texture feature	N0 (Mean ± SD)	N1-3 (Mean ± SD)
S(1,0)AngScMom	0.006 ± 0.002	0.004 ± 0.002
S(1,0)SumEntrp	1.73 ± 0.08	1.82 ± 0.07
S(1,0)Entropy	2.23 ± 0.178	2.53 ± 0.20
S(0,1)AngScMom	0.006 ± 0.002	0.003 ± 0.002
S(0,1)SumAverg	65.74 ± 1.17	64.69 ± 0.91
S(0,1)SumEntrp	1.72 ± 0.09	1.82 ± 0.07
S(0,1)Entropy	2.29 ± 0.18	2.53 ± 0.21
S(1,1)AngScMom	0.006 ± 0.002	0.004 ± 0.002
S(1,1)SumAverg	66.03 ± 1.60	64.90 vs. 1.23
S(1,1)SumEntrp	1.70 ± 0.09	1.80 ± 0.07
S(1,1)Entropy	2.32 ± 0.19	2.58 ± 0.23
S(1,-1)AngScMom	0.006 ± 0.003	0.004 ± 0.002
S(1,-1)SumAverg	65.88 ± 1.56	64.78 ± 1.33
S(1,-1)SumEntrp	1.70 ± 0.09	1.80 ± 0.08
S(1,-1)Entropy	2.31 ± 0.20	2.58 ± 0.24
S(2,0)AngScMom	0.006 ± 0.003	0.003 ± 0.002
S(2,0)SumEntrp	1.67 ± 0.10	1.78 ± 0.08
S(2,0)Entropy	2.31 ± 0.21	2.60 ± 0.25
S(0,2)AngScMom	0.006 ± 0.003	0.003 ± 0.002
S(0,2)SumAverg	66.15 ± 1.70	64.76 ± 1.35
S(0,2)SumEntrp	1.67 ± 0.10	1.77 ± 0.09
S(0,2)Entropy	2.31 ± 0.21	2.60 ± 0.26
S(2,2)AngScMom	0.007 ± 0.003	0.003 ± 0.002
S(2,2)SumEntrp	1.61 ± 0.12	1.74 ± 0.09
S(2,2)Entropy	2.27 ± 0.23	2.61 ± 0.27
S(2,-2)AngScMom	0.007 ± 0.004	0.004 ± 0.002
S(2,-2)SumEntrp	1.62 ± 0.11	1.72 ± 0.10
S(2,-2)Entropy	2.27 ± 0.23	2.59 ± 0.29
S(3,0)AngScMom	0.007 ± 0.003	0.003 ± 0.002
S(3,0)SumEntrp	1.61 ± 0.11	1.73 ± 0.90
S(3,0)Entropy	2.26 ± 0.23	2.60 ± 0.28
S(0,3)AngScMom	0.007 ± 0.003	0.004 ± 0.002
S(0,3)SumEntrp	1.61 ± 0.11	1.72 ± 0.10
S(0,3)Entropy	2.27 ± 0.23	2.59 ± 0.29
(3,3)AngScMom	0.008 ± 0.004	0.004 ± 0.002
S(3,3)SumEntrp	1.55 ± 0.14	1.69 ± 0.10
S(3,3)Entropy	2.21 ± 0.27	2.58 ± 0.30
S(3,-3)AngScMom	0.008 ± 0.005	0.004 ± 0.003
S(3,-3)SumEntrp	1.55 ± 0.14	1.67 ± 0.12
S(3,-3)Entropy	2.19 ± 0.27	2.55 ± 0.33
S(3,-3)DifEntrp	1.34 ± 0.11	1.42 ± 0.06
S(4,0)AngScMom	0.007 ± 0.004	0.004 ± 0.002
S(4,0)SumEntrp	1.57 ± 0.12	1.69 ± 0.10
S(4,0)Entropy	2.22 ± 0.25	2.58 ± 0.29
S(4,0)DifEntrp	1.34 ± 0.08	1.42 ± 0.05
S(0,4)AngScMom	0.008 ± 0.005	0.004 ± 0.003
S(0,4)SumEntrp	1.56 ± 0.14	1.68 ± 0.11
S(0,4)Entropy	2.22 ± 0.26	2.56 ± 0.32
S(4,4)AngScMom	0.01 ± 0.009	0.004 ± 0.003
S(4,4)Correlat	− 0.16 ± 0.24	0.026 ± 0.26
S(4,4)SumVarnc	171.20 ± 48.20	218.71 ± 61.00
S(4,4)SumEntrp	1.49 ± 0.17	1.67 ± 0.12
S(4,4)Entropy	2.10 ± 0.33	2.54 ± 0.32
S(4,-4)AngScMom	0.012 ± 0.013	0.005 ± 0.005
S(4,-4)SumEntrp	1.48 ± 0.21	1.64 ± 0.15
S(4,-4)Entropy	2.10 ± 0.34	2.49 ± 0.38
S(5,0)AngScMom	0.009 ± 0.006	0.004 ± 0.002
S(5,0)SumEntrp	1.53 ± 0.15	1.68 ± 0.10
S(5,0)Entropy	2.15 ± 0.29	2.56 ± 0.31
S(5,0)DifEntrp	1.34 ± 0.08	1.42 ± 0.05
S(0,5)AngScMom	0.010 ± 0.008	0.004 ± 0.004
S(0,5)SumVarnc	182.02 ± 63.31	225.87 ± 52.96
S(0,5)SumEntrp	1.51 ± 0.18	1.67 ± 0.12
S(0,5)Entropy	2.14 ± 0.30	2.53 ± 0.34
S(0,5)DifEntrp	1.35 ± 0.11	1.42 ± 0.06
S(5,5)AngScMom	0.026 ± 0.07	0.005 ± 0.004
S(5,5)Correlat	−0.24 ± 0.29	0.009 ± 0.24
S(5,5)SumVarnc	155.79 ± 62.01	214.60 ± 59.73
S(5,5)SumEntrp	1.38 ± 0.32	1.65 ± 0.14
S(5,5)Entropy	1.96 ± 0.46	2.48 ± 0.36
S(5,5)DifEntrp	1.26 ± 0.26	1.40 ± 0.08
S(5,-5)AngScMom	0.027 ± 0.070	0.007 ± 0.010
S(5,-5)SumEntrp	1.38 ± 0.34	1.60 ± 0.21
S(5,-5)Entropy	1.93 ± 0.47	2.42 ± 0.45
S(5,-5)DifEntrp	1.24 ± 0.26	1.39 ± 0.11
Horzl_RLNonUni	154.46 ± 85.77	519.4 ± 597.12
Horzl_GLevNonU	6.05 ± 2.79	18.84 ± 20.68
Vertl_RLNonUni	156.10 ± 87.25	526.97 ± 604.95
Vertl_GLevNonU	6.04 ± 2.81	18.94 ± 20.95
45dgr_RLNonUni	164.00 ± 88.86	562.36 ± 643.97
45dgr_GLevNonU	6.22 ± 2.83	19.41 ± 21.27
135dr_RLNonUni	165.72 ± 93.51	563.97 ± 643.96
135dr_GLevNonU	6.27 ± 2.92	19.47 ± 21.41
WavEnLL_s-1	15,263.48 ± 1237.94	16,505.73 ± 1013.61
WavEnLL_s-2	10,886.78 ± 2198.15	13,957.54 ± 2666.67

Underlined data are the statistically significant findings

**Fig. 4:**
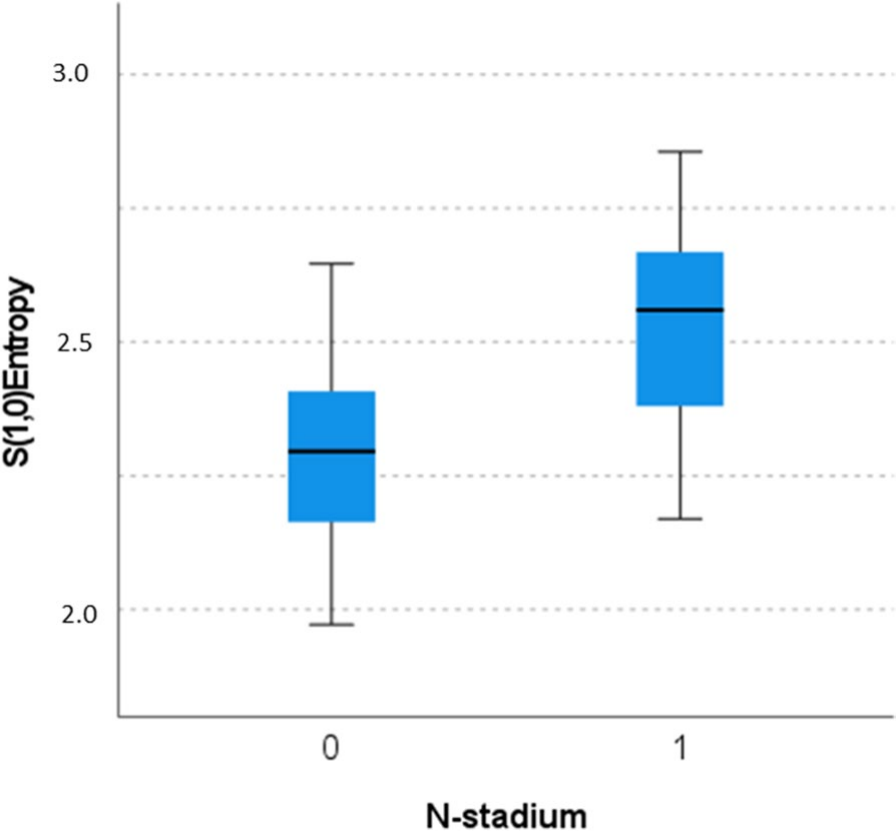
Discrimination analysis between N0 versus N1-3. The parameter “S(1,0)SumEntrp” reached statistical significance (Mann-Whitney test): 1.73 (IQR 0.12) versus 1.83 (IQR 0.11), *p* < 0.001

**Fig. 5:**
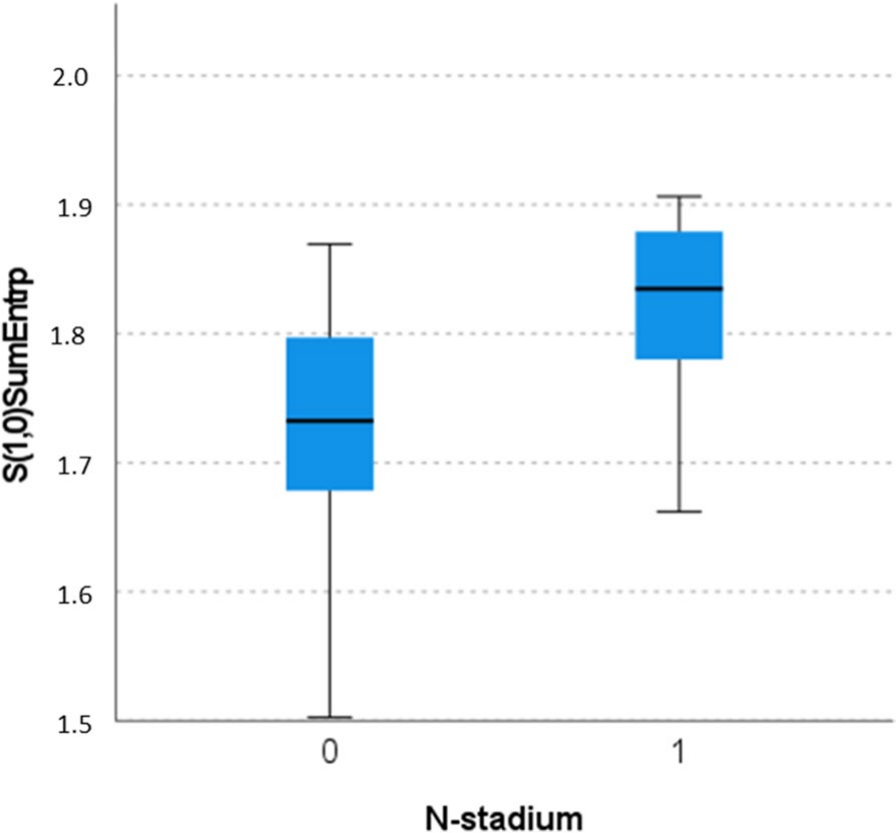
Discrimination analysis between N0 versus N1-3. The parameter “S(1,0)Entropy” reached statistical significance (Mann-Whitney test): mean 2.30 (IQR 0.26) versus 2.56 (0.30), *p* < 0.001

For instance, S(1,0)SumEntrp (1.73 ± 0.08 vs. 1.82 ± 0.07), as shown in Fig. 4, S(1,0)Entropy (2.23 ± 0.18 vs. 2.53 ± 0.20), as shown in Fig. 5, S(0,1)SumAverg (65.74 ± 1.17 vs. 64.69 ± 0.91) and S(3,-3)DifEntrp (1.34 ± 0.11 vs. 1.42 ± 0.06).

S(1,0)SumEntrp, S(1,0)Entropy and S(3,-3)DifEntrp were further investigated with ROC analysis.

**Fig. 6:**
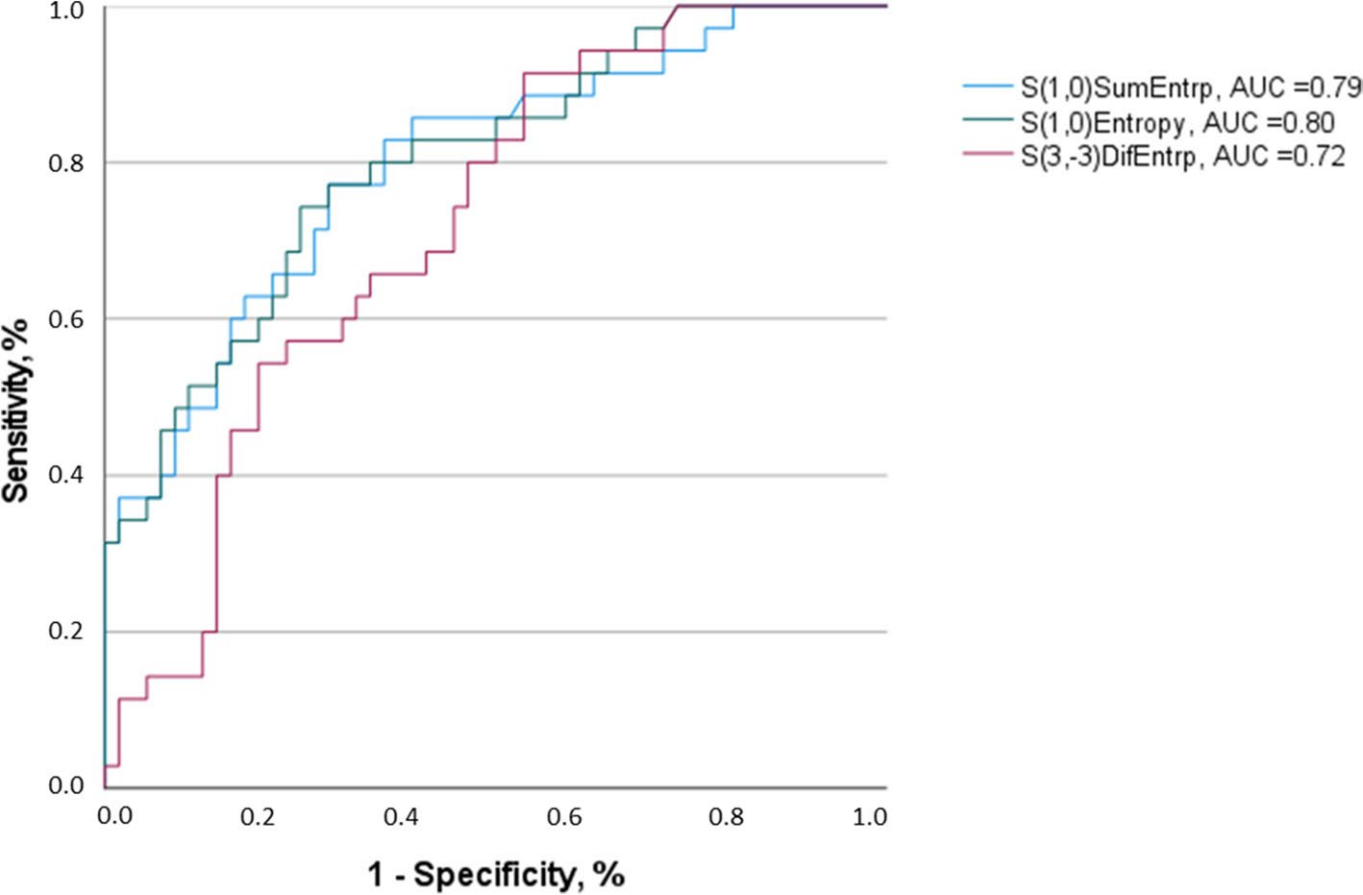
Results of the ROC-Analysis for discrimination of N0 vs N1-3 employing texture features S(1,0)SumEntrp, S(1,0)Entropy and S(3,-3)DifEntrp

For S(1,0)SumEntrp, an AUC of 0.79 was identified. A threshold value of 1.74 resulted in a sensitivity of 0.83 and a specificity of 0.64. For S(1,0)Entropy, an AUC of 0.80 was identified with a sensitivity of 0.80 and a specificity of 0.67 when using a threshold value of 2.35. For S(3,-3)DifEntrp, it achieved an AUC of 0.72 with a sensitivity of 0.66 and a specificity of 0.64 when utilizing a threshold value of 1.40. Figure 6 displays the corresponding graphs.

### Correlation analysis between texture features and total node-RADS score

In correlation analysis, the following correlating texture parameters were associated with Node-RADS score: S(4,0)Entropy (*r* = 0.72, *p* < 0.001), S(3,0) Entropy (*r* = 0.72, *p* < 0.001), S(2,2)Entropy (*r* = 0.72, *p* < 0.001), S(5,0)Entropy (*r* = 0.71, *p* < 0.001), S(0,2)Entropy (*r* = 0.70, *p* < 0.001), S(3,3)Entropy (*r* = 0.71, *p* < 0.001), S(2,0)Entropy (*r* = 0.70, *p* < 0.001), S(0,3)Entropy (*r* = 0.70, *p* < 0.001), S(4,4)Entropy (*r* = 0.70, *p* < 0.001), S(2,-2)Entropy (*r* = 0.69, *p* < 0.001), S(1,1)Entropy (*r* = 0.69, *p* < 0.001), S(0,4)Entropy (*r* = 0.69, *p* < 0.001) and WavEnLL_s-2 (*r* = 0.68, *p* < 0.001).

### Correlation analysis between texture features and node-RADS subcategories

#### Size

Short axis diameter showed statistically significant correlations with 28 texture features: r > 0.7 (*p* < 0.001): 45dgr_RLNonUni (*r* = 0.87), Vertl_RLNonUni (*r* = 0.86), 45dgr_GLevNonU (*r* = 0.86), Horzl_GLevNonU (*r* = 0.86), S(4,0)Entropy (*r* = 0.80), WavEnLL_s-2 (*r* = 0.72), S(1,0)SumEntrp (*r* = 0.70). A complete overview is given by Table 4.

**Table 4:** Significant correlations between texture features and short axis diameter of mediastinal lymph nodes with *p*-values < 0.001

Texture parameter	Spearman’s r
45dgr_RLNonUni	0.87
135dr_RLNonUni	0.87
Vertl_RLNonUni	0.86
Horzl_RLNonUni	0.86
45dgr_GLevNonU	0.86
135dr_GLevNonU	0.86
Horzl_GLevNonU	0.86
Vertl_GLevNonU	0.86
S(4,0)Entropy	0.80
S(2,2)Entropy	0.80
S(0,2)Entropy	0.80
S(3,0)Entropy	0.79
S(0,3)Entropy	0.79
S(5,0)Entropy	0.79
S(2,0)Entropy	0.79
S(2,2)Entropy	0.79
S(1,1)Entropy	0.78
S(0,1)Entropy	0.78
S(0,4)Entropy	0.78
S(1,-1)Entropy	0.78
S(3,-3)Entropy	0.77
S(0,5)Entropy	0.77
S(1,0)Entropy	0.76
S(4,-4)Entropy	0.74
S(5,5)Entropy	0.73
WavEnLL_s-2	0.72
S(1,0)SumEntrp	0.70
S(5,-5)Entropy	0.70

#### Texture

Texture subcategory showed statistically significant correlations with the following texture features (*p* < 0.001): WavEnLL_s-2 (*r* = 0.62), 45dgr_GLevNonU (*r* = 0.62), 135dr_GLevNonU (*r* = 0.62), Horzl_GLevNonU (*r* = 0.62), Vertl_GLevNonU (*r* = 0.61), 45dgr_RLNonUni (*r* = 0.60), 135dr_RLNonUni (*r* = 0.60), Vertl_RLNonUni (*r* = 0.60), Horzl_RLNonUni (*r* = 0.60), S(4,0)Entropy (*r* = 0.56), S(2,2)Entropy (*r* = 0.56), S(3,0)Entropy (*r* = 0.56) and S(5,0)Entropy (*r* = 0.55).

#### Border

Border subcategory showed statistically significant (*p* < 0.001) correlations with the following texture features: S(4,4)SumVarnc (*r* = 0.49), S(3,3)SumEntrp (*r* = 0.46), S(4,0)Entropy (*r* = 0.46), S(3,3)SumVarnc (*r* = 0.45) and S(3,3)Entropy (*r* = 0.45).

#### Shape

Shape subcategory showed statistically significant correlations with the following texture features (*p* < 0.001): Vertl_RLNonUni (*r* = 0.46), 45dgr_RLNonUni (*r* = 0.46), 135dr_RLNonUni (*r* = 0.46), Horzl_RLNonUni (*r* = 0.46), 45dgr_GLevNonU (*r* = 0.46), S(2,-2)Entropy (*r* = 0.46), Vertl_GLevNonU (*r* = 0.45), S(0,3)Entropy (*r* = 0.45), 35dr_GLevNonU(*r* = 0.45) and Horzl_GLevNonU (*r* = 0.45).

## Discussion

The present study sought to employ texture analysis as a quantitative assessment and Node-RADS as a semiquantitative assessment of mediastinal lymph nodes in patients with lung cancer. Key findings were that the two imaging assessment modalities were independently associated with both the presence of lymph node metastasis and with each other.

Texture analysis is an emergent field of research with extensive studies in several disease entities, predominantly in the field of oncologic imaging [1–7]. Clearly, several texture features derived from CT as well as MRI images are able to reflect distinctive histopathological tumor characteristics on a microstructure level, including lung cancer [1–7, 30, 31]. As such, in a recent study, the CT texture feature “CT texture joint entropy” and “CT entropy” were associated with hypoxia-related immunohistochemical features in head and neck cancer [31].

In a very promising multicentric study, CT texture analysis of the primary tumor was even able to predict the complex immune environment in lung cancer patients to guide treatment [32].

Therefore, the principal hypothesis of the present work was that texture analysis can also reflect relevant structural differences in mediastinal lymph nodes.

Several CT texture features were able to discriminate benign from malignant lymph nodes. Presumably, malignant lymph nodes show a higher heterogeneity due to tumor deposits resulting in a higher CT heterogeneity quantified particularly by entropy related second order statistics.

These findings are in line with the published literature. Koda et al. reported that the diagnostic possibility of CT texture analysis discriminated sarcoidosis lymph nodes from small-cell lung cancer with a very high sensitivity (100%), and specificity (92%), resulting with an AUC of 0.99 [18]. However, the results were based upon a small patient sample of 16 patients with sarcoidosis and 14 patients with small-cell lung cancer.

Another interesting study employed texture analysis on FDG-PET/CT images as a novel prognostic factor in patients with cancer of unknown primary [33].

In a study by Shin el al., CT texture analysis was utilized together with FDG-PET/CT images to predict the dignity of mediastinal lymph nodes in 80 patients with non-small cell lung cancer [19]. However, combinations of each item of chest CT and CT texture analysis did not achieve better diagnostic accuracy compared to the short axis diameter of 1 cm. Notably, only first-order texture features were utilized in this study, which might explain the differences to the present results.

In another study using „TexRad “package, only a sensitivity of 53% and a specificity of 97% were identified in the test sample for the investigated texture features [21].

In another study by Pham et al., a promising AUC of 0.89 were found based on regression analysis, and an accuracy of 70% based on the tenfold cross-validation. These results, which were retrieved from 133 malignant and 138 benign lymph nodes [20], were in line with the results presented herein.

Our results suggest that texture analysis might be employed in clinical routine to provide prognostic factors for clinical care in the work-up of mediastinal lymph nodes in patients with lung cancer.

Another aim of the present study was to validate the novel Node-RADS classification for the first time. Node-RADS is a novel proposed scoring system to categorize several imaging findings of lymph nodes on cross sectional images and to standardize their radiological reporting [8]. There is no restriction of this classification regarding the localization of the lymph node. In Node-RADS, a short axis diameter of 1 cm is proposed as general threshold. The present analysis revealed that Node-RADS is able to discriminate negative from positive lymph nodes. However, our results show a relatively high rate of malignancy even in the groups 1 and 2.

Another finding of the present study is that the proposed classification has only a moderate inter-reader agreement, which could limit the possibility for translation into clinical routine. This blends in the other proposed radiological classifications [34–38].

For BI-RADS, also only a moderate agreement for mammographic criteria was shown, even between experienced radiologists [34]. The inter-reader agreement for PI-RADS 2.0 scores was found to be only fair (kappa: 0.57, 95% confidence interval 0.49–0.66) [36]. The inter-reader agreement among all readers for the overall TI-RADS level was 50.4%, which is comparable to the present analysis [37].

This analysis provided a high malignancy rate, even in score group 2 (42.8%). However, the malignancy rate for BI-RADS 2 and PI-RADS should be 0%. In detail, the malignancy rate should strongly increase with the overall score. Thus, it is reported for BI-RADS 2 below 2%, for BI-RADS 3, 2-95% for BI-RADS 4, and 100% for BI-RADS 5 [39]. The malignancy rate for the Node-RADS groups 4 and 5 in the present analysis was 100% for both groups, which indicates that there might be no significant difference between the two categories in clinical routine.

Clearly, Node-RADS needs to be further validated in a larger sample size in terms of malignancy frequency.

There is a definite need that quantitative imaging aid in clinical decision-making process to enhance clinical care of cancer patients. Of date, the treatment of lung cancer patients has become more and more complex with an increasing number of molecular and prognostic markers. Imaging needs to address this fact with new biomarkers. The present study provides new insight into quantitative imaging of mediastinal lymph node analysis.

There are several limitations of the present study to address. First, it is a retrospective study with possibly known inherent bias. To reduce possible bias, texture analysis and Node-RADS scoring were performed in a blinded manner to the clinical and pathological results. Second, the patient sample size is relatively small due to the single center design and the time period considered. Moreover, the underlying primary tumors were heterogeneous, which could have influenced the results, since different primary tumors might have caused different texture results of the lymph nodes involved. Third, texture analysis still lacks standardization. There is a clear need to employ the texture features investigated in other patient cohorts scanned with different CT scanners to test for external validation of the present results. This is needed before the presented results can safely be translated into clinical care.

## Conclusions

Both CT-derived texture analysis and Node-RADS categorization can help discriminate mediastinal lymph nodes in patients with resected lung cancer who underwent lymphadenectomy. Both of the two imaging assessments can possibly be translated into clinical routine.

## Data Availability

The datasets generated and/or analysed during the current study are not publicly available due confidential information but are available from the corresponding author on reasonable request.

## Abbreviations

CT: Computed Tomography
AUC: Area under cure
ROC: Receiver-operating characteristics
RADS: Reporting and Data System
